# Neuromyelitis optica pathogenesis and aquaporin 4

**DOI:** 10.1186/1742-2094-5-22

**Published:** 2008-05-29

**Authors:** David J Graber, Michael Levy, Douglas Kerr, William F Wade

**Affiliations:** 1 Department of Pathology, Dartmouth Medical School, Lebanon, New Hampshire, USA; 2 Department of Neurology, Johns Hopkins Medical School, Baltimore, Maryland, USA; 3 Department of Microbiology and Immunology, Dartmouth Medical School, Lebanon, New Hampshire, USA

## Abstract

Neuromyelitis optica (NMO) is a severe, debilitating human disease that predominantly features immunopathology in the optic nerves and the spinal cord. An IgG1 autoantibody (NMO-IgG) that binds aquaporin 4 (AQP4) has been identified in the sera of a significant number of NMO patients, as well as in patients with two related neurologic conditions, bilateral optic neuritis (ON), and longitudinal extensive transverse myelitis (LETM), that are generally considered to lie within the NMO spectrum of diseases. NMO-IgG is not the only autoantibody found in NMO patient sera, but the correlation of pathology in central nervous system (CNS) with tissues that normally express high levels of AQP4 suggests NMO-IgG might be pathogenic. If this is the case, it is important to identify and understand the mechanism(s) whereby an immune response is induced against AQP4. This review focuses on open questions about the "events" that need to be understood to determine if AQP4 and NMO-IgG are involved in the pathogenesis of NMO. These questions include: 1) How might AQP4-specific T and B cells be primed by either CNS AQP4 or peripheral pools of AQP4? 2) Do the different AQP4-expressing tissues and perhaps the membrane structural organization of AQP4 influence NMO-IgG binding efficacy and thus pathogenesis? 3) Does prior infection, genetic predisposition, or underlying immune dysregulation contribute to a confluence of events which lead to NMO in select individuals? A small animal model of NMO is essential to demonstrate whether AQP4 is indeed the incipient autoantigen capable of inducing NMO-IgG formation and NMO. If the NMO model is consistent with the human disease, it can be used to examine how changes in AQP4 expression and blood-brain barrier (BBB) integrity, both of which can be regulated by CNS inflammation, contribute to inductive events for anti-AQP4-specific immune response. In this review, we identify reagents and experimental questions that need to be developed and addressed to enhance our understanding of the pathogenesis of NMO. Finally, dysregulation of tolerance associated with autoimmune disease appears to have a role in NMO. Animal models would allow manipulation of hormone levels, B cell growth factors, and other elements known to increase the penetrance of autoimmune disease. Thus an AQP4 animal model would provide a means to manipulate events which are now associated with NMO and thus demonstrate what set of events or multiplicity of events can push the anti-AQP4 response to be pathogenic.

## Introduction

### Neuromyelitis optica (NMO)

There are many excellent reviews on the clinical and laboratory aspects of NMO, reviews that describe criteria for diagnosis, and paraclinical features of NMO and the NMO spectrum of disorders [1-5]. We do not intend this to be a review of these issues. There is a complex, diverse array of "preceding environmental events" and perhaps unconnected immune-related events which are often associated with the period before patients are diagnosed with NMO. In this review we discuss in detail how the different isoform structures of AQP4 in different membrane locales and in different cell types might be related to pathology. Changes in AQP4 expression in CNS and non-CNS tissue can be regulated by inflammatory mediators induced during and following infection or by underlying autoimmunity and can result in the induction of AQP4-specific lymphocytes and ensuing pathogenesis.

NMO is a devastating disease affecting primarily young women (relapsing NMO) but either sex can develop monophasic NMO, and NMO rarely occurs in adolescents. The disease principally attacks the optic nerves and spinal cord causing blindness and paralysis. The most notable difference between NMO and multiple sclerosis (MS) is the lower frequency of brain lesions in NMO, especially early in the disease [6]. An autoantibody (IgG1) that binds AQP4 has been found in a high percentage (~75%) of NMO patients (NMO-IgG) [7]. NMO patients that do not have detectable levels of NMO-IgG1 may represent a group for which AQP 4 is not the target antigen for autoantibody. Other CNS antigens such as the Kir4.1 present on astrocytes might be targets for autoantibodies in those NMO patients. It is possible that there is a unique and rare specificity of NMO-IgG1 that is particularly pathogenic but that can not be detected by current diagnostic techniques – mouse tissue to screen NMO sera. Clearly to address these issues the NMO-IgG1 concentration, epitope specificity, and affinity need to be better categorized at the initial presentation and during the patient's response to treatment.

Other autoantibodies have been found in NMO patient sera and CSF, including antinuclear antibodies, SS antibodies [8] and in particular anti-myelin oligodendrocyte glycoprotein (MOG) antibodies [9,10]. Other antibodies specific for extra- or intracellular antigens (myelin basic protein, S100β, CPSF-73, RNF-141, and myosin light chain are also present in some NMO patients. These latter autoantibodies likely represent a response to neo-antigen liberated from dead cells and thus are not the initial cause of NMO but could be involved in the pathogenesis of recurrent disease via a type III hypersensitivity reaction [9,11]. Consistent with the potential role of a humoral response in NMO pathogenesis is the perivascular deposition of IgM and IgG, both of unknown specificity. In addition, the terminal products of complement (C9) activation are found with antibody depositions in spinal cord lesions of NMO patients [12].

### AQP4 a transmembrane protein important for CNS function

Aquaporin 4 is a type III transmembrane protein (intracellular n- and c-termini) that regulates water entry into and out of specific cells in the brain and interfaces with blood vessels within the neuropil and around the ventricles. The AQPs and the aquaglyceroporin mammalian family contains at least 12 members that are involved in water and/or glycerol transport [13] but AQP4 is the predominant water channel in the brain of humans and rodents. The more than 200 different AQP-like "sequences" expressed by diverse organisms underscores the importance of this protein family [14]. AQP4 regulates water mostly in multiple types of tissue epithelia, but it also has a critical role when expressed by astrocytes which regulate water and ion movement in parts of the brain.

The expression pattern of AQP4 protein is most well characterized for rats which express AQP4 in multiple tissues and in specific cell populations in particular tissues (Table 1). In humans, expression of AQP4 in the brain, spinal cord, and optic nerves is associated with astrocyte membranes that closely appose endothelial cell basal membranes. It is of note that astrocytes interact extensively with endothelial cells to maintain the CNS BBB, which normally limits the access of immune system effectors unless localized or distant events disrupt the BBB, thus allowing access of cellular or soluble immune effectors. The BBB is maintained by active interactions between astrocytes and endothelial cells. There are regions in the brain that lack these close interaction based on anatomical organization of the two cell types, and in these areas the BBB is compromised. These regions include termini of peripheral nerves (olfactory), sensory ganglia (spinal and cranial nerves), and the optic nerves. Some ependyma and some capillaries in the CNS lack continuous endothelia with tight junctions [15].

**Table 1:** Tissues with AQ4 expression organized via biological system.

**System ***Animal (references)*	**AQ4-Positive Tissues ***(Detection Methods)*^*a*^	**Positive Cell Types**	**Cellular Localization**	**Expression Pattern**
**Nervous ***human [68, 135] mouse [136, 137] Ra*t *[16, 19, 55, 135, 138–140]*	Gray and White Matter *(IHC, IEM)*	Astrocytes	Perivascular End-Feet	Orthogonal Arrays
	Ventricles, but not in Choroid Plexus *(IHC, IEM)*	Ependymal Cells	Basolateral	Orthogonal Arrays
	Pia Mater *(IHC)*	Meningeal Cells	N.D^b^	N.D.
	Retina *(ISH, IF, IEM)*	Müller Cells Astrocytes	Vitreal End-Feet Perivascular End-Feet	N.D.
	Posterior Optic Nerve *(ISH, IF, IEM)*	Astrocytes	Perivascular End-Feet	N.D.
	Inner Ear *(ISH, IF, IEM)*	Hensen's Cells Inner Sulcus Cells Supporting Cells of the Vestibular End Organ	Basolateral	N.D.
**Urinary ***mouse [137] rat [138, 139, 141]*	Kidney Medullary Collecting Duct *(IEM, IB, IHC, IF)*	Epithelial Cells	Basolateral	Orthogonal Arrays
**Respiratory ***human [142] rat [138]*	Ciliated Ducts of Bronchi *(IF, IHC)*	Columnar Epithelial Cells Intermediate cells	Basolateral	N.D.
	Acini of Bronchi Submucosal Glands *(ISH)*	Epithelial Cells	N.D.	N.D.
	Trachea *(IHC)*	Epithelial Cells	Basolateral	N.D.
**Muscular ***human [143] mouse [137, 143] rat [139, 143]*	Skeletal Muscle *(IF, IEM, RT-PCR)*	Fast-Twitch Fiber	Sarcolemma	Limited Orthogonal Arrays
**Digestive ***human [144] Rat [138, 139, 145]*	Stomach *(IHC)*	Parietal Cells Chief Cells	Basolateral	N.D.
	Villi of Distal Large Intestine *(IHC)*	Epithelial Cells	Basolateral	N.D.
	Salivary Gland Excretory Duct *(IHC)*	Epithelial Cells	Basolateral	N.D.
	Liver Bile Duct *(RT-PCR, IB)*	Epithelial Cells	Basolateral	N.D.
**Integumentary ***mouse [146] rat [139]*	Lacrimal Gland and Excretory Duct *(IHC, IF)*	Glandular Cells Epithelial Cells	Basolateral	N.D.

^**a**. ^IF: immunofluorescence, IEM: immunoelectro microscopy, IHC: immunohistochemistry, RT-PCR: real-time polymerase chain reaction, IB: immunoblot

^**b**.^N. D., not determined

AQP4 is expressed in astrocytes in the neocortex, hippocampus, cerebellum and many circumventricular structures including the glial lamellae of the supraoptic nucleus and the subfornical osmosensory organ of the brain [16]. The microvessels of the subfornical organ have a caudal to rostral difference in the BBB due to the distinct association of astrocytes with endothelial cells at different parts of this organ [17]. AQP4 is also expressed in a subpopulation of CNS ependymal cells associated with the pia, subfornical organ, and to a lesser extent in other ependyma but not in the choroid plexus. In the hypothalamic nuclei and in the subfornical organ, AQP4 is not localized to one aspect of the membrane, but rather distributed [16]. AQP4 is expressed *in situ *in LPS-activated microglial cell (monocytic origin) membranes [18]. While not technically in the CNS, retinal astrocytes (Müller cells) have polarized expression of AQP4 with high levels seen in end-feet membranes that oppose the vitreous body or blood vessels but either reduced or no expression on non-end-feet membranes and microvilli, respectively [19]. Orthogonal arrays of intramembranouse particles composed of AQP4 are expressed in fibrous astrocytes that associate with optic nerves [19]. The distribution of AQP4 is compartmentalized with end-feet membranes having high expression, while the membranes facing the nodal axolemma have lower expression. In the perinodal processes, AQP4 is preferentially expressed in the membrane opposite from the nodal membranes. AQP4 is also expressed in the optic nerve head which is unmyelinated.

### Different isoforms of AQP4

**Figure 1:**
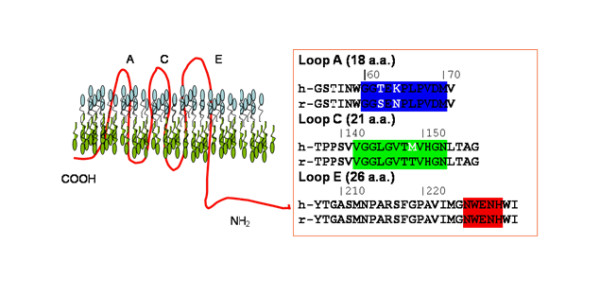
**Aquaporin 4 is a type III water channel regulator with limited surface exposed residues**. AQP4 has been cloned from mice, rats, and humans. Algorithms predicted a type III transmembrane protein with intracellular n- and c-termini (left panel shows topology). Resides predicted in the extracellular loops are shown in one letter code. Residues based on the crystal structure [25] that are predicted to be in the exposed extracellular loops are highlighted in either blue (Loop A) green (Loop C), or orange-red (Loop E). Differences between rat (same as mouse) and human sequences in the exposed loops are noted by single letter code in white.

Aquaporin 4 exists as two major isoforms (M1 and M23) in rat and human astrocytes and some epithelial tissues [20-22]. The M1 and M23 isoforms differ by 23 amino acids at the N-terminus depending on which methionine is used to start translation [20]. Based on algorithm analysis of the AQP4 sequence, the membrane topology of AQP4 has been proposed [23] to have three extracellular loops (A, C, and E) that connect the 6 alpha helices that span the membrane (Figure 1). Freeze-fracture data of AQP4-containing membranes, along with the identification of AQP4-specific antibodies in NMO patients that bind extracellular AQP4 support the notion that parts of AQP4 are accessible on the outside of the cell [7,21,24]. Recently, the structure of rat AQP4 has been more extensively analyzed and a model based on electron crystallography of double-layered, two-dimensional crystals has been proposed [25]. The crystal structure of AQP4 suggests, in contrast to the algorithm analysis, that Loop E is more intramembranous and involved in water transport [25].

Depending on the cell type, AQP4 can be expressed on basolateral, apical or at both membrane locations with the AQP4 isoform and protein-protein associations dictating the localization. In CNS astrocytes, AQP4 is associated with transmembrane proteins, α and β – dystroglycan (α-dystroglycan can bind laminin), Dp 71 (a dystrophin isoform), and syntrophin [26]. Deletion of α-dystroglycan did not reduce AQP4 expression in all end-feet membranes, as AQP4 labeling was retained in the end-feet that impinge on the subarachnoidal space and in cells that line the ventricles. These results differ from those obtained using the *mdx *mice (dystrophin-null lacking all splice forms) that do not express AQP4 in subarachnoid end-feet or cells that line the ventricles [27,28]. Deletion of α-syntrophin results in loss of AQP4 expression in astrocyte end-feet but not in AQP4 expressing cells in the kidney or osmosensitive regions of the brain [26]. α-dystroglycan and laminin interactions are proposed to anchor the astrocytic end-feet (Müller cells) to their perivascular location in retina [29]. Laminin interacts with dystroglycan, a component of the multi-protein complex which, in rat astrocytes, is localized at the same membrane regions as aggregates of the membrane channel protein Kir4.1 (potassium inward rectifying) and AQP4 (M23 isoform) [30].

Complex transmembrane, cytoskeletal and extracellular matrix components (agrin, heparan sulfate proteoglycan; laminin, extracellular matrix protein) regulate access of AQP4 to the apical domain of astrocytes where different lipid species and additional membrane proteins (e.g., K^+ ^leak channels) share the "domain space" with complex arrays of AQP4. These complex associations are considered essential for AQP4 function in astrocytes [31,32]. The genetic ablation of α-syntrophin is not fatal to mice but the loss of expression does influence AQP4 expression in the CNS. AQP4 expression in endothelial cells (not a major pool of AQP4 expression) is maintained while the AQP4 in astrocyte end feet is lost [33].

A recent study indicated AQP4 was internalized following overnight culture at 37°c. with NMO-IGG serum [34]. The significance of this observation to pathogenesis of NMO disease is moderated by the use of only the M1 isoform in HEK cells that do not normally express AQP4. The influence on the architecture of AQP4 complexes by M23 and other protein elements needs to be understood before the role of anti-AQP4 antibody-mediated internalization of M1 AQP4 can be linked to NMO pathogenesis [30,35,36]. It was recently reported that the presence of cysteine residues and thus the potential post translational changes (palmitoylation) in M1 residues 13 and 17 of the N-terminus of AQP4 prevents formation of AQP4 arrays [37].

### AQP4 homotetramers and arrays

The homotetramer structure for AQP4 is consistent with the structure of other AQPs [20,25]. While AQP4 cannot associate with other AQPs, it can form heterotetramers of M1 and M23 [20]. The importance of the M1:M23 isoform ratio to function *in situ *is not known, but in cultured epithelial cells, large M23-based arrays were more efficient at water transport than M1 AQP4 [38]. All of the heterotetramers of M1 and M23 are equal in water transporting characteristics when expressed in *Xenopus *oocytes [20]. The isoform ratio of AQP4 greatly influences the orthogonal arrays of the AQP4 tetramers [21,39]. The M23 isoform is associated with 4–6 nM intramembranous particles in arrays as large as 100 particles. In contrast, M1 tetramers, which are the same size as M23, are overwhelmingly (95%) found as single tetramers [39]. In the tetramer model, individual AQP4 subunits are proposed to interact with each other via residues (R108, G157, W231 I239, Y250) in the H4 and H6 helices [25]. Quaternary interactions are possible between AQP4 molecules within tetramers or between AQP4 tetramers on apposing membranes thus potentially limiting access of some AQP4 regions to AQP4-specific antibodies.

### AQP4 structure, immunogenicity, and B cell epitopes

The initial HEK transfectants employed to define NMO-IgG binding used "full-length" human AQP4 which is presumably the M1 isoform [7] that does not form arrays in Chinese hamster ovary or epithelial cells [21,39]. Two other studies by Takahashi and colleagues [40,41] also used HEK cells expressing an unidentified human AQP4 isoform and supported the previous report of Lennon and co-workers. This might have been a fortuitous design allowing the identification of anti-AQP4-specific antibodies in NMO patient's sera. Alternatively, the use of M1 might overestimate the ability of anti-AQP4-specific antibodies to bind to structures composed of the M23 AQP4 isoform which is the predominant form found in astrocytic end-foot processes [42]. One study reported the use of human AQP4 M23-transfected HEK cells to assess sera from Japanese MS patients with optic-spinal and longitudinal spinal cord lesions [43]. The AQP4 staining in this study was positive and intense for rat CNS tissue with sera that Tanaka and co-workers considered positive for NMO-IgG, but it was different in intensity and staining pattern compared to the confocal images of Lennon and colleagues [7]. However, images with control sera were not available so the issue of signal to noise could not be assessed. In support of the capability of NMO-IgG to bind arrays of AQP4 *in situ *are the early studies of NMO patient sera that demonstrated positive staining of murine CNS tissue [24] using fluorescence microscopy which does not distinguish between isoforms of AQP4. Based on the quaternary structure of AQP4 *in situ *and its interactions with other membrane structures it is difficult to envision a model whereby all potential B cell epitopes would have equal access to extracellular NMO-IgG. Expression of AQP4 on the basolateral cell membranes versus apical cell membrane could also moderate the availability of AQP4 targets for immune system effectors, especially AQP4-specific antibodies.

### Optic nerve and AQP4 distribution

Several studies have reported that in NMO, areas of CNS inflammation correlate with the expression pattern of AQP4 [44,45]. Classically, NMO targets the spinal cord and optic nerves and spares the brain. However, since the development of the NMO-IgG test, case reports of NMO patients with lesions in the brain have expanded the clinical criteria of NMO to include lesions in the brainstem, deep grey matter and cerebellum, areas that have relatively high expression of AQP4 [46,47].

The access of serum components to CNS tissues is generally limited, but there are distinct anatomical locations of the CNS where the BBB has reduced capability to moderate serum access. AQP4 is expressed by astrocytes that surround the optic nerve. The optic nerve head is an area of the CNS where the BBB is more permissive [48]. It has been noted that while patients with NMO can develop ON and LETM simultaneously, they are generally displaced in time with ON being experienced first and LETM presenting weeks to months later [8]. Is there something about the tissue organization of the optic nerve cells that express AQP4, the vascular structures associated with the optic nerves, or the type of astrocytes (fibrous astrocytes) associated with the optic nerves that result in ON as first indicator of AQP4-specific antibody pathology?

The prelaminar region of the optic nerve head is an area of the CNS that has a permeable BBB as evidenced by immunostaining for markers of intact BBB [49]. Tracer studies using rhesus monkeys demonstrated that blood-borne proteins can reach optic nerve axons through the border tissue of Elschnig in the optic nerve head [50]. In this study, access to the optic nerve head was localized at a barrier formed by glial cells in the tissue of Kuhnt at the edge of the optic disc that prevented tracer access to the subretinal space. AQP4 is expressed in fibrous astrocytes that impinge on optic nerves preferentially in the membrane that face away from the nodal membranes. The localization of AQP4 on fibrous astrocytes might function to spare the optic nerve from volume fluctuation under normal physiological conditions. Perhaps one of the reasons that ON with anti-AQP4-specific antibodies might initially be more prevalent than LETM in NMO is that the tissues of the optic nerve are more sensitive to volume changes induced by AQP4 dysfunction than other areas of the CNS [19]. Tso and co-workers [50] also noted that the pia and fibrous septa of the optic nerve showed no leakage. Thus if inflammation in NMO is related to accessibility of serum components across the BBB, the optic nerve head should be preferentially affected compared to other parts of the optic nerve.

### Spinal cord and AQP4 distribution

Analysis of AQP4 distribution in the human and rodent spinal cord has been of major interest since the discovery of the NMO-IgG. Aquaporin 4 (mainly the M23 variant) is expressed in ependyma and astrocytes in association with blood vessels or nerves [20,51] in both the gray and white matter of rat spinal cord [51,52] and in glial cell processes adjacent to the ependymal cells of the central canal. In the white matter, fibrous astrocyte processes that surround blood vessels are also positive for AQP4. The AQP4 expressed in the spinal cord differs from other nervous tissue cells as it is rarely polarized in astrocytes. AQP4 was most strongly present in fibrous astrocytes of the two most superficial lamenae (substantia gelatinosa) of the dorsal horn of the spinal cord, but less noticeable in the intermediate gray matter and in the ventral horn [51,52].

In NMO, the cervical and the upper thoracic spinal cord segments are generally more affected and single longitudinally extensive lesions starting in the cervical spine and reaching into the brainstem are thought to be specific for NMO. The cervical cord is closest to the cervical lymph nodes where priming AQP4-specific responses is likely to occur. One animal model proposes that the cervical lymph nodes play a major role in the transverse myelitis associated with EAE [53]. It is unclear if similar mechanisms play a role in the pathogenesis of NMO.

### Ependyma and AQP4 distribution

The ependyma is another region of the BBB where the diffusion of serum components into the CNS is possible. In rodent and human brain, AQP4 is expressed in basolateral domains of ependymal cells lining the ventricles and central canal of the spinal cord [52]. Ventricular ependyma has been reported to more highly express AQP4 than ependyma lining the central canal of the spinal cord [54]. Ependymal cells have direct access to CSF as they do not have a basement membrane and do not express tight junctions, thus AQP4-specific antibodies in the CSF would have relatively free access to their target [55]. However, when anti-AQP4 antibodies are detected in the CSF of NMO patients, the concentration is less than 1:500 compared to the serum. Astrocytic foot processes along the pial lining also express high levels of AQP4. In a recent review of NMO by Pittock [56], regions in the pia were noted to have pathologic changes in seemingly random areas. It is of interest to understand how the difference in AQP4 expression of ependyma and pia-associated astrocytes contributes to the potential pathogenic role of NMO-IgG.

### Potential AQP4 B cell epitopes and relationship to pathogenesis

**Figure 2:**
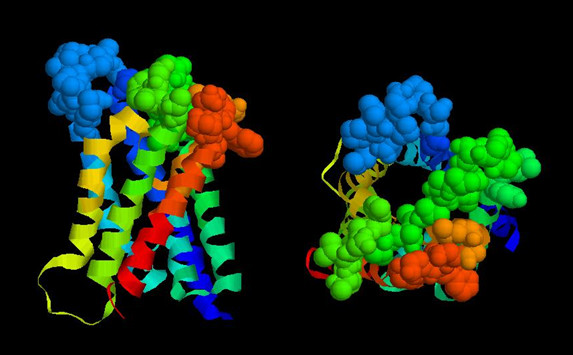
**Model of rat AQP4**. The RasMol 2.7.2 program is available on the WWW and was used to manipulate the protein data base file, 2D57 [25]. RasMol 2.7.2 is an updated version of RasMol 2.6 developed by Roger Sayle while at the Biomolecular Structures Group, Glaxo Wellcome Research & Development, Stevenage, Hertfordshire, UK. The left image shows both ribbon and space filling motifs. The ribbons are the six alpha helices that span the plasma membrane. The c-terminal and n-terminal domains were not part of the crystal structure but would be projecting downward from the red and blue helices, respectively. The top of the molecule shown in space filling format represents three loops that are thought to be surface exposed: Loop A (blue), Loop C (green) and Loop E (orange-red). The image to the right is the view of the top of AQP4 looking down its central axis.

**Figure 3:**
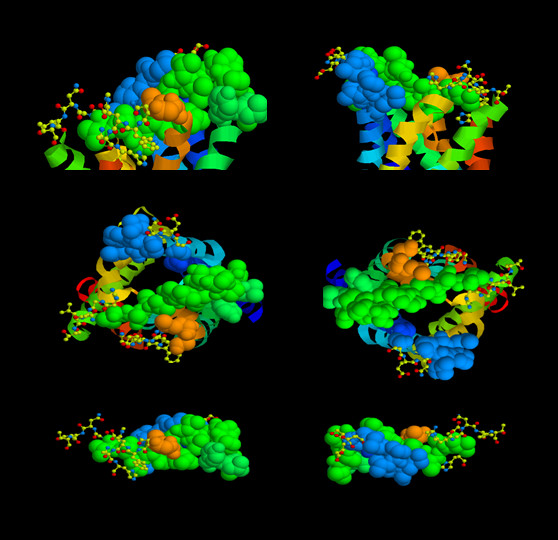
**Potential B cell epitopes of AQP4**. The top two panels represent a side view (left image, Loop E facing viewer; right image Loop A to the left) of AQP4 with amino acid residues shown in helical, space-filling, or ball and stick format. The space-filling format represents the proposed extracellular loops (Loop A, blue, Loop C green, and Loop E orange-red). Ball and stick motif represent amino acids within the loops that are potential antibody contact residues because of O (red) or N (blue) atoms available for H-bonding or salt-bridge interactions with anti-AQP4-specific IgG1. The middle panel is the top view of AQP4 with formatting the same as described for the top two images. The bottom set of images has been restricted to show only the loops. This view clearly shows the lateral projection rather than upward along the long axis of AQP4 of side-chains of amino acids that could interact with anti-AQP4-specific antibodies (left image, Loop E facing the viewer; right image, Loop A facing viewer). The amino acids in ball and stick representation are: Loop A, S62, E63, N64; Loop C, H151, N153, T155; Loop E, N226, E228, H229.

B cell epitopes are molecular signatures, structures that fit in the antigen binding site of antibodies. The antigen binding site of antibodies is composed of structural contributions from the antibody's heavy and light chains. The variable immunoglobulin gene exons with the intrinsic first and second hyper-variable regions along with the third hyper-variable region, formed by the junction of the variable, diversity, and joining sequence, can either be in germ line (baseline binding affinity) configuration or can have undergone somatic mutations that are selected for improved binding to its B cell epitope. Antibody binding sites can vary in their structure and can accommodate small molecules or large protein such that the binding site surface area can range from several hundreds to over a thousand square angstroms. Resolution of the AQP4 structure provides evidence that both small defined B cell epitopes and extended conformation epitopes are possible based on exposed amino acids (Figure 2). The surface area of the top of the AQP4 molecule is on the order of 35 × 30 Å^2^, a size that could represent a conformational epitope with contributions from several exposed loops of AQP4. The extracellular loops that connect the various helices of the AQP4 water channel are thought to be exposed. However, Loop C, the longest loop, contains many non-polar residues and the structure as presented by Hiraoki and coworkers [25] is modeled as being recessed in the center of the complex. The other loops, A and E are smaller and contain several residues that are hydrophilic and typical partners for H-bonding or salt bridge interactions that are important for antibodies binding affinity. At issue is how many of the residues and if their corresponding site chains are exposed "around" the top of AQP4 as potential B cell epitopes required for NMO-IgG binding (Figure 3).

**Figure 4:**
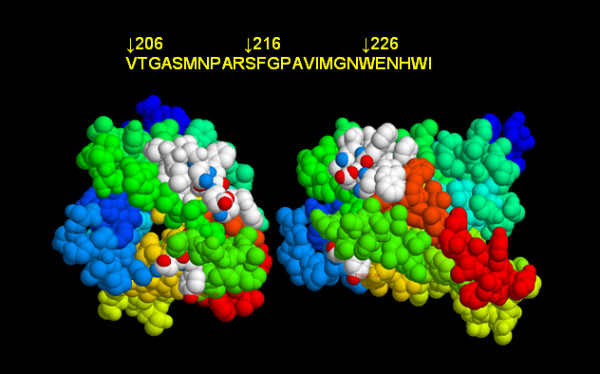
**Immunogenic peptide of AQP4**. The space filling motif on the left shows the helices from the top of the AQP4 molecule. The white residues are amino acids 206–231 with several shown in CPK format that highlights oxygen (red) or nitrogen (blue) atoms that might be available for interactions with antibody combining sites. The residues in white on the bottom of the figure are 207, 208 while those on the top represent 216–231 with 224–229 in CPK format. In residues 224–229, M224, G225, W227, and E228 for a cluster of residues that have multiple atoms available for H-bonding. The image on the right is rotated 90° relative to the image on the left.

An initial mapping of NMO-IgG may provide some clues as to the AQP4-associated B cell epitopes. Lennon and co-workers used mouse tissue as an antigen source for immunohistochemistry [24]. NMO-IgG binds multiple sites including the abluminal face of microvessels, pia, subpia, and Virchow-Robin sheath of fixed murine CNS sections. This is important as the AQP4 sequence between mice, rats, and humans differs within the exposed residues in the middle of Loop A [25,57]. In mice and rats, Loop A has 62S, 63E, and 64N, whereas in humans the residues are 62T, 63E, and 64K. Thus the binding of human NMO-IgG to murine AQP4 suggests at least three interpretations: 1) that Loop A is not a B cell epitope, 2) the changes between rodents and humans do not affect antibody binding if the loop A sequence is a B cell epitope., or 3) the sequence changes do affect binding of anti-AQP4 antibodies, but other AQP4 epitopes are still available and antibodies in polyclonal sera have enough reactivity to AQP4 to be scored as positive for binding if one AQP4 epitope is compromised. The second interpretation would suggest more defined linear sequences as B cell epitopes that would be expressed in the A or E loop structures. Other information on possible AQP4 B cell epitopes was reported by Nagy and colleagues [58] who made monoclonal antibodies to rat AQP4 peptides. An IgM of low affinity was made to residues 206–231 (VRGASMNPARSFGPAVIMGNWENHWI) which form part of the water channel helix and Loop E (Figure 4). The AQP4 crystal model suggests that only the N- and C-termini of the peptide 206–231 are surface exposed and thus residues able to form a B cell epitope might be limiting (Figure 4). This B cell epitope is largely located on the lateral side of the AQP4 model and thus accessibility concerns *in situ *need to be addressed. We have recently demonstrated that antibodies raised in mice to this peptide bind human M1 and rat M23-based arrays of AQP4 in transfected HEK cells (unpublished observations, WF, Wade).

Clonal expansion of higher affinity AQP4-specific B cells and activation or immune deviation due to AQP4-specific T helper (Th) cells might reflect the time interval between ON and LETM. Does affinity maturation of the anti-AQP4 (B cell or T cell) response have to occur for AQP4-specifc antibodies to bind more inaccessible arrays of AQP4 on astrocyte foot processes in the spinal cord? Perhaps ON can be more readily induced by lower levels of effectors, NMO-IgG and AQP4-specific T cells as fibrous astrocytes cell might be more sensitive to anti-AQP4 antibody than astrocytes in the spinal cord.

In the MOG-mouse TCR transgenic model of ON a related question is apparent – why do MOG-specific transgenic TCR mice only developed ON and not EAE. Interestingly, treatments to induce changes in the BBB increase the manifestation of ON, but not of EAE, suggesting that the access of MOG-activated T cell to the spinal cord or brain is not sufficient to cause EAE. Bettelli and coworkers [59], who developed the MOG-ON model, speculated on the role different concentrations of the autoantigen peptide, the type of autoimmune peptide, the background genetics, and tissue localization of the target antigen in explaining the localized inflammation in this model. Other MOG-models readily induce EAE indicating MOG-specific T cells can be pathogenic.

### Pathology of human NMO – then and now

There are multiple descriptions in the 19^th ^century medical literature of the clinical manifestations of patients with a disease characterized by unilateral or bilateral loss of vision and myelitis [1]. Devic and Gault are credited with the development of the criteria for a diagnosis of Devic's disease. The early Devic's diagnostic criteria based on autopsy were as follows: a macroscopically normal brain with severe demyelination in the spinal cord and optic nerves, cavitating lesions in the white or gray matter, necrosis, blood vessel wall thickening, lymphocyte infiltration, but no significant neutrophil or eosinophil infiltration, which were only later observed by recent autopsy of NMO patients [12,60]. One must be careful of over interpreting the early literature because of the potential for multiple causal events that resulted in either a clinical or autopsy diagnosis of Devic's disease. The limited number of reported case studies is also problematic for determining the extent of the clinical picture of Devic's disease in the last two centuries.

Since the discovery of the association between NMO-IgG and Devic's disease, NMO has replaced the name of Devic's disease and the criteria have been refined and extended to include assessment of MRI imaging of the spinal cord and brain [61]. MRI of the brain should be inconsistent with multiple sclerosis and MRI of the spine should show a contiguous extensive lesion more than 3 vertebral segments in length in the setting of myelitis. In addition, the presence of NMO-IgG was added as an additional supportive criterion for a diagnosis of NMO [62], but is not required for the diagnosis.

The recent NMO literature indicates NMO can be either a fulminating monophasic disease (20%) or more typically one that is polyphasic (80%), with long term survival being related to the number of attacks in the early period after the initial event. Current therapies include glucocorticoids, azathioprine, plasmapheresis, intravenous immunoglobulin, and anti-CD20 monoclonal antibody treatment [63-67]. It is unclear how aggressive modern drug treatments along with plasma exchange and immunosuppression have modified the natural course of disease. Therapies that are effective in NMO patients are those that, modify inflammation (gene activation events), reduce lymphocyte proliferation, remove serum immunoglobulin and other components and in the case of the anti-CD20 monoclonal antibody, kill pre-B cells, mature B cells, and plasmablasts, but not lymphocyte stems cells or plasma cells.

Biopsy and post-mortem autopsy pathology shows a characteristic pattern in CNS tissue of patients with NMO. In addition to demyelination, NMO lesions are necrotic and infiltrated with neutrophils and eosinophils. Complement and immunoglobulin deposits of types IgM and IgG are found in a vasculocentric pattern that mirrors that expression pattern of AQP4 in normal appearing white matter [12]. Unlike MS in which AQP4 expression is up-regulated in actively demyelinating plaques, NMO lesions show a defining loss of AQP4 immunoreactivity. Even in patches of so-called normal appearing white matter where there is no other evidence of inflammation, AQP4 expression has been shown to be reduced. These characteristics of NMO lesions are typical of all NMO lesions including spinal cord and optic nerve most commonly, followed by brainstem, cerebellum and cerebral cortex [68].

Another aspect of the modern diagnosis of NMO is the analysis of CSF. Case reports and retrospective studies indicate that oligoclonal banding can be found in NMO patients, but at a much lower frequency than that found in MS patients. The intrathecal IgG also tend to disappear as NMO patients progress in treatment or disease [69,70]. Another feature of intrathecal antibodies of MS patients is the tendency to have polyclonal specific responses to viral antigens which is rarely found in NMO patient CSF [71].

It would be of interest to know if increased pleocytosis noted in the more recent clinical work-ups was also present in patients in the late 1800s and early 1900s [72]. Although detection of NMO-IgG in CSF is rare, CSF of NMO patients, on average 4 years into what was characterized as aggressive disease, were found to contain anti-MOG antibody (IgM and IgG) cells, as well as mononuclear IL-5, IL6 secreting cells [10]. Eotaxin-2 and Eotaxin-3, which are selective chemotactic factors for eosinophils, and eosinophil cationic protein were higher in NMO patient CSF than in normal CSF. The elevation of these molecules was not specific to NMO as other neurologic conditions have elevated eosinophil chemotactic factors in the CSF.

### NMO pathogenesis

Wingerchuck and co-workers at the Mayo Clinic proposed a model that featured the anti-AQP4 antibody and how it might be pathogenic [5]. They indicated that the pathogenic anti-AQP4 antibody binds to the extracellular component of the AQP4 protein and induces a reversible internalization of the AQP4-IgG complex. The immunopathology aspect of the model is well-conceived and possible. In this model, the authors leave out the role of AQP4-specific B and T cells which certainly are part of the immune response to AQP4.

The incomplete nature of negative selection of self-reactive B cells in the bone marrow or induction of anergy in the peripheral B cell pool can result in a very high percentage of the B cell in the periphery with the capacity to secrete autoantibodies. It is very likely that the normal human B cell repertoire includes AQP4-specific B cells. B cells can be non-specifically activated by infection or inflammatory conditions that provide the cytokine milieu and membrane proteins which can sustain B cell survival and which can enhance their survival and selection. If these cells enter the germinal center, they can undergo somatic mutation and isotype switching of their low affinity IgM to high affinity IgG. The predisposition toward autoimmunity is also associated with conditions that allow survival of B cells with unwanted specificities [73].

Thus given the multiple means to push an existing B cell repertoire to secrete autoantibodies (including NMO-IgG), it is necessary to have a better understanding of the tissue expression and the regulation of AQP4 in those tissues so that initiating and amplifying events in AQP4-specific T and B cell activation can be followed. The other reason to understand this issue is related to the potential pathogenicity of NMO-IgG antibodies which is probably limited by the isotype, as IgM and IgA have more limited access to tissue parenchyma than IgG. Further, not all cells that express AQP4 exhibit pathologic changes in NMO patients. The reason for this is not known, but may be related to the access of expressed AQP4 in organs and tissues such as in kidney and gut epithelia to serum components or the association of AQP4 with other membrane proteins.

### HLA-restriction, AQP-4 specific T cells, and NMO

NMO is not a common (1%) demyelinating disease of Caucasians being more common in East Asians and other non-white populations [74]. There is a current debate as to whether NMO and Asian MS are the same disease. The finding of NMO-IgG in a large percentage of Asian MS patients substantiates this perspective [24,43,75]. The genetic link for these differences is not known but if presentation of self antigen is part of the mechanism leading to NMO, MHC genotype would be important and perhaps a distinguishing feature. This genetic link may be linked to other factors which influence B cell survival, cytokine production, MHC-based antigen presentation, or target antigen regulation.

It seems reasonable to hypothesize that AQP4 specific T cells are present in patients with NMO-IgG as AQP4 is a protein antigen that requires T cell help for induction of AQP4-specific B cells. In addition, the isotype (IgG1) of AQP4 antibodies is associated with germinal center reactions, another component of an immune response that involves T cells. A MHC class II linkage (HLA, DPA1*0202; DPB1 0501; reactive with myelin basic protein) has been identified in patients (90%) with Asian MS that some consider NMO; however the high expression of this HLA type in Asians tempers the initial interpretation of a causal link between HLA type and disease [76]. The cognate antigen for the Asian MS group was mainly MOG, with some reactivity to other oligodendrocyte antigens. AQP4 T cells were not examined by the screening method. Another group reported the severity of debilitation of NMO patients correlates with the increased frequency of Vβ 7 and Vβ 13 [one of the chains forming the antigen specific T cell receptor (TCR)] expressing T cells in CSF of NMO patients [77]. A close examination of ON and LETM patients for HLA type and AQP4-reactive T cells should be a priority. If they are present, then it is important to know if the T cell effector phenotypes mediate lesion differences (ON and LETM versus NMO). It would be interesting to know if ON and LETM patients who do not go on to develop NMO have a different AQP4-specific TCR and T helper (Th) cell profile than those who develop NMO.

### Priming of AQP4-specific B and T cells

A universal tenet of the immune response to MHC-associated antigen (self or non-self) is that antigen needs to be recognized in the context of co-stimulation, a requirement for priming antigen-specific T cells. Initial recognition and priming of lymphocytes specific for protein antigens is accomplished by professional antigen presenting cells (APC) such as, dendritic cells (DC), B cells, and macrophages. Immune responses are dependent on lymphocytes that express antigen-specific or antigen-crossreactive TCR and BCR interacting with the APC they encounter while trafficking in the secondary lymphoid tissue. Priming is the initial recognition that results in clonal expansion of naïve B and T cells and then effector and memory cells. During infection, the type of cell death (apoptosis or necrosis) of AQP4-expressing cells is important for priming AQP4-specific-T cells. If carrier epitopes (TCR epitopes) are provided by pathogen proteins, inflammation with polyclonal conditions could enhance the AQP4 specific B cells entry and maintenance in the memory pool.

The tissue source of AQP4 might be important for the efficiency and frequency of priming of AQP4 specific B and T cells. AQP4 is expressed within and outside the CNS of humans, rats, and mice (Table 1). There is cell death in the CNS and trafficking of DC-like cells within the CNS resulting in tissue debris draining ultimately into cervical lymph nodes [78,79]. Microglial cells are also known to express class II restriction elements and present antigen in a manner that can moderate or enhance a pathogenic immune response. Their role in priming naïve AQP-4-specific T cells in draining lymph nodes is thought to be minimal, but they could clearly interact with AQP4-specific T cells at the site of NMO pathology [80,81]. CNS antigens can enter the periphery by drainage through tissue below the cribriform plate and into the nasal mucosa [82,83]. Alternatively, perivascular CNS macrophages that survey the CSF for soluble and tissue debris from effete membrane and exocytic processes can migrate to CNS draining lymph nodes [81,82]. Migration of CNS macrophages to cervical lymph nodes results in presentation of the peptide components of the gathered CNS species in association with MHC class II molecules resulting in maintenance of peripheral tolerance of T cells if co-stimulation is not provided [83].

The hosts' genetics (HLA selection of T cell repertoire) coupled with infectious disease or inflammatory history, would contribute to events which either favor or not the initiating events of AQP4-specific lymphocyte activation. As AQP4 reactive B and T cells traffic between lymph nodes, the initial priming could occur at either CNS-draining or non-CNS draining lymph nodes, but once activated the normal steady state of presentation of autoantigens by multiple APC (DC and macrophages associated with the perivascular space, leptomeninges or choroids plexus) microglia, or astrocytes could activate T cells as they traffic in the CNS. Previous activation of T cells in CNS draining lymph nodes could amplify the AQP4 reactive lymphocytes and their ability to traffic into the CNS. Activated AQP4-specific T cells would cause disruption of the BBB thus allowing increased concentration of AQP4 antibodies and other immune system effectors to enter the tissues where AQP4 is expressed on astrocyte apical membranes. Further activation of the AQP4-specific T cells even in low numbers could sustain the BBB breach and recruitment of effectors that cause more pathology with time.

Bacterial and viral infection can damage respiratory epithelial cells which express AQP4, thus providing conditions to enhance antigen presentation by mature dendritic cells as well as obviating the influence of regulator T cells which could otherwise play a role in the typical non-pathogenic responsive to AQP4. Because the potential for simultaneous expression of AQP4 peptides, pathogen-associated peptides, and TLR agonists is possible the T cells that provide help for AQP4-specific B cells, the T cells do not have to be AQP-4 specific and could be pathogen-specific T cells. Another means to activate AQP4-specific B cells is bystander activation of AQP4-reactve B cells by the cytokine milieu and associate membrane changes of dendritic cells during infection or autoimmunity that enhance B cell survival and differentiation. Once activated, AQP4-specific B cells are very effective antigen presenting cells and could be the important step in the subsequent activate of AQP4-specific T cells via BCR-internalized AQP4.

The scenario of AQP4-specific activation of B and T cells is similar in autoimmune diseases. Salivary and lacrimal glands express autoantigens associated with SS and can also be disrupted by infection or autoimmune attack. As salivary glands express AQP4, the initiating autoimmune response to non-AQP4 antigens might liberate AQP4 that because of intrinsic failures in immune regulation in autoimmunity would facilitate AQP4 B cell activation and survival.

Infection and autoimmunity can result in events that alter the BBB and thus permit access of AQP4-specific antibodies and T cells to the CNS. Some cytokines, universal regulators of cells of the immune system, can affect the BBB as well as the expression of glial cell-associated AQP4. Interleukin 1β and interferon β up-regulate AQP on rat astrocytes [84], whereas interferon γ(IFN-γ) but not Interleukin 1β or TNF-α increases expression of human astrocyte AQP4 *in vivo *[85]. The interaction between glial and endothelial cells is critical for BBB function [86,87]. Conditions which activate these cells and cause upregulation of AQP4 when the BBB function is compromised would result in higher epitope density for anti-AQP4-specific antibody binding that could disrupt function of any one of the cells and thus lead to changes in water retention which may be part of the initial pathology of NMO. It would be interesting to determine if antibodies to Kir4.1, an inward-rectifying potassium channel co-localized with AQP4 on astrocyte foot processes are able to cause NMO-like disease.

### Does the Th cell have to be specific for AQP4 in order to induce AQP4-specific antibodies

It is generally accepted that activated-antigen specific T cells can enter the brain and cause disruption of the BBB for CNS autoimmune disease. There are at least two antigens for which specific T cells might be relevant for NMO, as both MOG and AQP4 antibodies have been detected in CSF and sera of NMO patients. The CNS cells that express MOG and those that express AQP4 differ. MOG or AQP4-specific T cells enter the CNS and would recognize cognate antigen presented by endogenous by APC in the CNS at multiple locations. Microglial are capable of "cross-presenting" MOG and presenting endogenous AQP4. Macrophages and DC are associated with the perivasculature of the pia which also contains AQP4-expressing astrocytes. Thus there are at least two possible incipient antigens that might be primary in the induction of NMO.

This scenario raises an important point: Is NMO a specific disease (target and type of pathology) due to a specific antigen such as AQP4? Alternatively, is NMO a syndrome with distinct but multiple causes triggered by multiple distinct antigens that induce autoreactive T cells and high affinity antibodies? These immune effectors might react with several exposed CNS antigens and cause tissue damage or disruption of the astrocyte/glial axis that set the stage for long term immune effector access and disease. The answer to whether NMO is a specific disease, a syndrome, or subtype of MS awaits the identification of the insipient autoantigens for both disease phenotypes.

### Epigenetic events-major or minor role in NMO

Is there something special about certain types of neural inflammation and subsequent expression of cellular antigens in the context of co-stimulatory molecules at the astrocyte/endothelial cell interface resulting in anti-AQP4-specific B cells whereby only a limited set of individuals go on to develop NMO? In the syndrome hypothesis above, it is likely that AQP4-specific B cells are readily induced in certain infections but NMO does not result, or is self-limiting as is typical for pediatric NMO patients. Subsequent resolution of the infection and tissue damage limits AQP4 exposure at non-CNS draining lymph nodes and prevents the activation of AQP4-specific autoimmune effector cells. Are recurrent or specific infections near the neck and head where the draining cervical lymph nodes have a higher concentration of AQP4 protein more likely to be the priming event for ON or LETM? Once AQP4-specific B and T cells are activated, how does the constitutive expression of AQP at leaky BBB contribute to pathogenesis? An animal model is required to address these types of questions.

### Potential pathogenic role of AQP4-specific antibodies

The presence of myelin basic protein specific antibodies, as well as other autoantibodies, in cord blood suggests that the human immune system has multiple clones of B cells early in life that could contribute to autoimmunity [88]. The human B cell repertoire (germline) has B cell clones that express a BCR reactive to AQP4 either as a *bona fide *antigen or as a cross reactive species. NMO-IgG can be associated with younger individuals [89,90] but is more typically found in older individuals in their fourth decade of life. Pathology associated with bacterial or viral infections can target organs/tissues that express AQP4 and thus prime AQP4-reactive B cells if they are not clonally deleted or anergized during tolerance induction.

Further studies are needed to prove the pathogenic role of anti-AQP4 IgG1, whether it be singular, additive or synergistic with other antigen-specific effectors of the immune system. NMO, ON, and LETM patients' B cells should be positively selected and either immortalized or cloned as a source of cells to screen for anti-AQP4 antibodies. IgM, as well as isotype-switched antibodies specific for naturally expressed AQP4, should be evaluated in more patients with autoimmunity and NMO. The generation of these types of reagents early in the disease presentation and during the progression of the disease would have several benefits. The nature of the Ig locus in human AQP4-specific B cells could be evaluated and it could be determined whether the anti-AQP4 IgG1 have germline sequences or have somatically mutated sequences. If the latter occurs, then the B cells have likely undergone germinal center reactions signifying interaction with antigen-specific T cells. Correlation of the affinity of anti-AQP4 IgG1 and the B cell epitope targets of those antibodies on AQP4 should be useful as a means to further evaluate disease progression or prognosis.

The presence of IgM, IgG, and complement cascade products in the pathology of NMO optic nerve and spinal cord may represent non-specific antibodies as well as anti-AQP4-specific antibodies. Human monoclonal AQP4-specific IgG1 antibodies should be used to generate anti-idiotype reagents that could be used to identify AQP4 IgG1 binding *in situ *of autopsy tissue. A practical advantage of the anti-idiotype reagent if they are of high enough affinity would be imaging of patients tissues while they are experiencing clinical symptoms as well as during the period of remittance of the clinical disease. As a complement to determining how or if, anti-AQP4 IgG1 contributes to NMO pathology; anti-AQP4 antibodies need to be evaluated with respect to their affinity, epitope specificity, isotype or subclass, and ultimately pathogenic potential. We need to know if a specific concentration or specificity of anti-AQP4 antibodies is required to manifest NMO. Similarly, we should try to understand the role of AQP4-specific T cells in inducing inflammatory disease of the optic nerve and spinal cord.

### NMO in association with other diseases

NMO-IgG has been found in patients with various infections and autoimmune disorders, as well as neoplastic, conditions not typically consistent with induction of the immune system against CNS AQP4. The autoimmune diseases associated with NMO can be either organ specific (Myasthenia Gravis, MG) or systemic (Systemic Lupus Erythematosus, SLE) but have a common characteristic of the involvement of autoantibodies specific for a panel of extracellular and intracellular antigens. Several reports of thymoma (without Herpes simplex virus -2 involvement) related NMO-like disease has been reported. One was associated with anti-CV2/CRMP5 antibodies and another MG [91,92].

### Autoimmune disorders and NMO

Clinical signs of NMO or NMO-like disease have been reported coincident with [93,94] or following symptoms of SS [95,96]. Sjögren's Syndrome is an autoimmune disease characterized by inadequate tear and saliva production and a lymphocyte infiltration of exocrine glands, especially the lacrimal and salivary glands, which express AQP 5 on the apical membrane and AQP4 on lateral membranes [97,98]. Sjögren's Syndrome patients typically have autoantibodies that bind the nuclear antigens and RNA-binding proteins, Ro52, Ro60, and La48. In SLE, immune complexes deposition can affect the skin, joints, kidneys, lungs, nervous system, serous membranes and/or other organs of the body whereby inflammation damages membranes (lungs, kidney, nervous system) which also can contain AQP4. Autoantibodies associated with SLE typically bind ssDNA or dsDNA, DNA-associated proteins (PCNA and histones), snRNAs, ribosomal P, and phospholipids. Clinical signs of NMO have been diagnosed together with SLE [99,100] or years later [101,102]. Most of the reported cases of SLE associated with NMO were prior to the identification and use of NMO-IgG1 as a diagnostic tool. NMO-IgG1 is detected in most sera of patients with concomitant NMO or NMO partial syndromes together with SS or SLE, but not in sera of SLE or SS patients that are not diagnosed with NMO [8,103]. In the study by Pittock and co-workers, the limited patient base who had SS or SLE, or both diseases predated the NMO diagnosis by 1–8 years with a bimodal distribution of 1 year or ≥ 7 years [104]. They concluded that patients with other autoimmune disorders who also test positive for the NMO-IgG have manifestations of NMO superimposed on their baseline autoimmune disorder. It will be important to accrue more patients for these types of analyses to see if progression is either fast or slow and if this relates to different types of anti-AQP4 antibody responses.

Myasthenia gravis is an autoimmune disease that features antibodies that bind the acetylcholine receptor in muscle motor neural endplates thereby causing weakness (ocular, bulbar, limb), and respiratory distress. It is interesting the same multi-protein complex that associates with AQP4 and localized it to the membrane in astrocytes is also involved with aggregation and localization of the acetylcholine receptor at the neuromuscular junction [105,106]. Myasthenia gravis is the prototypic neuroimmunologic disease caused by a pathogenic antibody. Autoimmune autonomic neuropathy is another neurologic disease that is likely to be mediated by a pathogenic antibody [107] and NMO may be the third. An antibody is classified as pathogenic if it meets 5 specific criteria [108]. First, the antibodies should be present in a substantial fraction of patients with the disease. In studies of the incidence of NMO-IgG in NMO, the range varies from 30–75%. Second, the antibody should bind to a specific target. NMO-IgG has been shown to bind to AQP4 and confirmed by several labs. Third, passive transfer of NMO-IgG should reproduce features of the disease. Recall the elegant experiments in MG where passive transfer of human anti-acetylcholine receptor antibody caused disease in mice [109]. This has not been demonstrated in NMO. Fourth, immunization with the relevant autoantigen produces a model disease in animals. This also has not yet been demonstrated in NMO. Fifth, reduction of the antibody should ameliorate disease. To date there has been only a single study demonstrating the relationship of titer to disease activity in an NMO patient [41].

While T cells are likely involved in providing help for production of autoantibodies, T-cell effectors are not associated with the pathology at postsynaptic neuromuscular junctions [110], supporting the prospect that anti-AQP4-specific antibodies could be pathogenic by themselves. Cases of NMO diagnosed after onset of MG often had surgical intervention for thymoma [91] or thymus hyperplagia [111,112]. Forty percent of MG patients who were evaluated for NMO-IgG were positive and two female patients with NMO and MG were of Japanese ancestry [111,112]. There is also a unique case report of an individual diagnosed with MG and NMO together years after onset of SLE in addition to Grave's disease [112].

### Infections and NMO-IgG

Many NMO patients report a preceding bacterial or viral infection of different tissues, especially epithelial tissues, prior to the onset of neurologic symptoms. NMO following pulmonary tuberculosis in patients from Cape Town, South Africa [113-116] or mycoplasma pulmonary infections [117,118] have also been reported. Mycobacterium and Mycoplasma sp. are known to express proteins that have homology to AQPs [119]. A possible link has been suggested between mycoplasma infections and TNFα, eicosanoids, and nitrous oxide triggered by Mycoplasma sp. stimulated rat glial cells [120].

We searched the protein data base for protein homology of the extracellular loops of AQP4. We found aquaporins that Mycobacterium and Mycoplasma sp. express that have similar residues to human AQP4 (unpublished observations, Wade WF). Syphilis was thought to be causal in a case report of and NMO without anti-AQP-4-specific antibody [121]. In many of the cases of parainfectious NMO the presence of AQP4-specific antibodies is not reported and some CSF profiles are not consistent with NMO. However in some reports, MRI analysis (spinal cord) and clinical presentation are consistent with a diagnosis of NMO.

Viral infections can often lead to ON. While not common, NMO has been associated with HIV-1 infection in an African woman [122]. Another viral disease that can precede NMO is dengue fever, which is prevalent in Brazil. NMO or NMO-like symptoms following within a week or so of viral infection was reported in a young female patient of Japanese ancestry, while a 58-year-old man of Japanese ancestry who also lived in Brazil was admitted to hospital suffering from acute disseminated encephalomyelitis (ADEM) after Dengue virus infection [123,124]. Several different types of herpes infections (Cytomegalovirus, Varicella) have been reported in patients with apparent NMO [125-127]. The Cytomegalovirus patient who was negative for NMO-IgG was a male who switched gender, but it was not reported if hormone therapy was in place. Female sex hormones are known to be able to affect B cell biology and thus enhance the possibility of autoantibody production [128]. Both of the Varicella patients were female, one black and one Bangladeshi whose ethnicity was not reported. With the new criteria for NMO it would be very useful to track such infection related cases with respect to anti-AQP4-specific antibody at the time of presentation after recovery or at autopsy. HLA-tissue typing of the rare cases of infections that lead to NMO would be of value as well.

### Animal models of NMO-like diseases

Currently there are no animal models that feature AQP4 as the autoantigen that induces NMO-like pathology. It is important to initiate development of an AQP4-based animal model of NMO to determine whether AQP4 is the autoantigen required for NMO, either due to AQP4-specific T and B cells, specific AQP4 antibody or both cellular and soluble effectors. We recently induced high titer antiserum in mice to the rat 206–231 sequence of the E Loop of AQP4 (unpublished observation, Wade WF). Pooled sera from the AQP4-peptide immunized mice bind the M1 human isoform and the rat M23 isoform of AQP4 in HEK cells. Similarly, an AQP4 peptide (QTKGSYMEVEDNRSQVETED, amino acids 272–291) from rat AQP4's cytoplasmic domain, that is predicted to be an autoimmune T cell target, was immunogenic in rats (unpublished observation, Graber DJ, Hickey WF). Investigators at Johns Hopkins University have induced high titers of autoantibodies to extra- and intracellular AQP4 peptides in Lewis rats although it has been more difficult in C57 mice (unpublished observation, Levy M, Kerr D). Thus the target sequences within AQP4 with which to investigate NMO are beginning to be realized and thus soon will be available to develop AQP4 animal model based on those enumerated and developed for MOG-induced CNS autoimmunity.

There are multiple rodent (mice or rat) models of EAE which can be induced with myelin antigens. The value of the animal EAE models which are based more on the pathology of MS than NMO is the mechanistic information they can provide. One of these models demonstrates that the presence of spinal cord pathology can be dependent on the presence of CNS antigen-specific autoantibodies [129,130]. The induction of these antibodies can be regulated by the form of the autoantigen or the genetics of the host. Tissues that are the targets of pathology in EAE and NMO can overlap but the details of the immune response and the autoantigen can shape the final effector phases. The differences in EAE and NMO pathology should not be used to eliminate the investigation of an EAE-NMO model system that might highlight common mechanisms relative to antigen-specific T cells and changes in the BBB that can enhance the pathogenic potential of autoantibodies.

In two rodent models of MOG-induced disease, there is NMO-like pathology implying that an AQP4-based system could be developed [129-131]. The similarity of the AQP4 protein sequence between humans and these rodents, and the fact that human NMO-IgG binds murine CNS AQP4 further substantiates the feasibility of developing an AQP4-based rodent model of NMO. In the case of mice, the established method of generating transgenic animals with AQP4-specific BCR and TCR would be a benefit. The availability of various immune mice that have select immune regulators genetically deleted (knockout mice) would help define the parameters that can be enhance the penetrance of the disease as it is only partial in the murine double, transgenic MOG-antigen receptor mice. Rats have long been used as CNS autoimmune models in part because of the size of their target organ, which along with the well-described role of rat AQP4 in rat CNS physiology would be an argument for using rat as an experimental host. The other advantage of the BN strain for the basis of an AQP4 NMO model is the disease manifests (60%) within weeks of inoculation and importantly, NMO-like pathology can be induced by the naive B and T cell repertoire.

### Mouse model – MOG-specific BCR transgenic

There is a murine model wherein the mice spontaneously develop ON [59]. This model is based on transgenic expression of a TCR specific for MOG, peptide 35–55. Transgenic MOG-specific CD4 T cells, without prior antigen activation causes ON in 30% of the mice. Immunization of mice with MOG 35–55 peptide (10 or 100 μgs) in complete Freund's adjuvant without pertussis toxin enhanced the percentage (55% and 78%, respectively) of mice with ON [59]. The MOG peptide can activate CD4 T cells and is long enough to contain B cell epitopes. However, neither the presence of anti-MOG 35–55 antibodies in these mice nor that of Anti-AQP4 antibodies was reported.

A later study that used the MOG 35–55 specific TCR transgenic mice examined the retinal ganglion cell damage induced by spontaneous or pertussis toxin-induced ON [132]. This study extended the first report by showing that pertussis toxin enhanced the development of ON. Further this group showed that inflammation preceded retinal cell loss, suggesting that increased access of T cells to antigen or activation of MOG-specific T cells or both in the optic nerve resulting in lesion development in a larger percentage of mice. An issue that was not addressed by either study was which cell type was the *in situ *APC (microglia, macrophages or dendritic cells) in the optic nerve tissue. CD4 T cells are typically restricted to MHC class II which is not normally expressed on oligodendrocytes, the cell type that expressed MOG.

### MOG-specific BCR and TCR double transgenic mice

Bettelli and coworkers [129] and Krishnamoorthy and colleagues [130] modified the MOG TCR model by crossing these animals to a line of animals with a rearranged B cell receptor (BCR) specific for a conformational epitope of MOG. In human MS, MOG-specific antibodies can be either specific for conformational or linear determinants, but the conformational antibodies are those associated with pathology [133]. Mice that express both a BCR and a TCR specific for MOG epitopes develop inflammation of the spinal cord. BCR MOG-specific transgenic mice or those mice crossed with a non-MOG specific TCR transgenic mouse line that have MOG specific IgM do not have NMO-like pathology [130]. The co-expression of MOG-specific BCR and MOG-TCR transgenic lymphocytes results in isotype switching of the MOG antibodies to IgG1, which in mouse is related to a different Th-profile than that associated with the human AQP4 IgG1 subclass. In the double transgenic, IgG1 and not IgM is correlated with disease. Increased activation of MOG-specific T cells was thought to be due to enhanced antigen presentation by MOG-specific B cells, while the role of isotype-switching was not investigated [130]. The CNS pathology of the double transgenic was proposed to be NMO-like because the lesions were in the optic nerve and spinal cord as seen in NMO patients. However the perivascular IgM, IgG, and complement products associated with human NMO were not present in the double transgenic model, nor were anti-AQP4 antibodies [130]. The inflammatory cell types seen in NMO, mainly eosinophils with some neutrophils, [10,12] were generally absent in the cellular infiltrates reported by Krishnamoorthy and coworkers [130]. Thus the double MOG transgenic mice do not reproduce NMO pathology but do provide a clear link to understanding how antigen specific B cells can play a role in regulating the pathogenicity of autoreactive T cells.

### Rat model-antibodies to MOG are important

The Brown Norway (BN) rat EAE model is particularly instructive for thinking about the pathogenesis of NMO. Immunization of BN rats with recombinant MOG protein but not MOG peptides results in EAE [131]. In BN rats anti-MOG specific T cells are not pathogenic compared to Lewis rats. One of the reasons for this is thought to be the type of Th response. A Th 2 response is more likely in BN rats than in Lewis rats, which have fulminate EAE after immunization with MOG peptide and adjuvant [134]. The differences in the genetics that may cause this are beginning to be unraveled and MHC as well as other intragenic MHC or linked genes, may play a role. Of special note is that in BN rats, the manifestation of EAE is dependent on high titers of antibody to MOG. Either by passive or active immunization both T cells and B cells specific for MOG result in immunopathology in the spinal cord and optic nerves characterized by the accumulation of neutrophils and eosinophils.

### Approachable questions based on a small animal NMO model

It seems clear that AQP4-specific T cells are activated in NMO. Lymphocyte accumulation in the lesion area of early NMO patients were not characterized, but the fact that activated T cells are a major cause of changes in the BBB and the fact that T cell help is required for isotype-switching to protein antigens, suggest that AQP4 specific T-cells are present. What they do in NMO is not known and might be only important in isotype switching. Expression of class II molecules is a potential issue that might mediate the choice or rats over mice, as rats express class II on non-professional antigen presenting cells in a similar manner to humans. We do not know how efficient murine and rat MHC class II are at binding AQP4 peptides and activating AQP-specific T cells. The activation of T cells is dependent on the type of APC, the cytokines they can make, and the MHC/peptide density, both of which can be reflected in the Th-profile of the T cells that are selected and expanded. The quality of antigen presentation is also important for selection of most fit (higher affinity for MHC/peptide) antigen-specific T cells. If the rodent MHC molecules are limiting, then transgenic mice expressing human HLA DPA1 0202/DPB1 0501 might be required to induce T cells that respond to AQP4 and thus cause disease. Clearly the types of experiments that investigate the role of both T and B cells AQP4-specific effectors will be important.

Once the AQP4 model is established, investigations into the parameters that can affect disease manifestations will be decisive in understanding events that can contribute to humans developing NMO. Experimental designs to address these questions are not approachable in humans. An AQP4 small animal model will permit investigators to examine the following experimental questions: 1) What peripheral or CNS "event" can lead to anti-AQP4 immune responses, and 2) what AQP4 specific immune responses are shaped by infection or underlying autoimmunity that result in NMO? 3) Has the current therapy for NMO changed the immunophenotype of the pathology? 4) Does anti-CD20 therapy which removes anti-AQP4 specific B cells but does not quantitatively deplete AQP4-specific antibodies work because AQP4-specific T cells are no longer activated?

It would be very interesting to determine if a particular isotype of rodent anti-AQP4 antibody (IgG2a in the mouse is equivalent to human IgG1) is pathogenic. The isotype may not be as relevant for pathogenesis as the antibody's concentration and affinity which could be easily tested in the rats with monoclonal reagents. The role of anti-AQP4 specific antibodies and B cells versus the individual components could be tested with passive transfer experiments. Immunization of AQP4 into mice with underlying immune dysfunction (enhanced B cell activating factor expression, mutations in CD95 or its ligand) might be a useful approach to study enhanced survival of AQP4-specific B cells and thus to determine if NMO disease manifestation is more frequent.

## Conclusion

The available data does not show a clearly defined mechanism explaining the association of NMO-IgG and NMO. The current paradigm of CNS autoimmunity includes activated, antigen-specific T cells as the principle means to moderate the BBB. It is critical to determine if there is a pathogenic role for NMO-IgG in NMO. A potentially unique mechanism whereby anti-AQP4 antibodies initiate pathology by themselves is that they have access to certain areas of the BBB that are considered more permeable and thus can initiate damage to a critical 'gatekeeper" (astrocytes) that normally preclude access of other immune components to the CNS. AQP4 expression is elevated in many conditions of CNS inflammation and diseases such as spongiform encephalopathy, Alzheimer's, stroke and others which do not have an infection or autoimmune component. Perhaps the importance of AQP4 for regulating brain edema suggests that when its function is interrupted and the BBB is compromised, immunopathology ensues. Several different but perhaps limited specificities of autoantibodies targeting antigens at specific sites (optic nerve and spinal cord) high in AQP4 expression and a structural BBB that is intrinsically more permissive to serum proteins may be required for NMO. If the autoantibodies are not pathogenic *per se*, it may be that the ability of self-reactive B cells to capture their cognate antigen and, in the case of AQP4 or MOG, present antigen to normally non-reactive MOG or AQP4-specific T cells, instigates NMO. There are multiple pathways whereby AQP4 from peripheral and CNS sources can induce AQP4-specific T and B cells. Inflammation and underlying autoimmune dysfunction can enhance AQP4 expression, prime antigen-specific lymphocytes, and establish conditions to sustain autoimmune responses. An AQP4-based animal model needs to be developed to address these issues and determine if AQP4-specific antibodies represent a novel means to cause CNS immunopathology similar to NMO disease in humans. We need to know if AQP4-specific T cells are dispensable or if they are part of a more complex scheme of pathogenesis that includes anti-AQP4-specific antibody which is consistent with the general paradigm of antigen-specific T cells being important to CNS autoimmunity.

## List of abbreviations

APC: antigen presenting cell; AQP4: aquaporin 4; BBB: blood brain barrier; BN rat: Brown Norway rat; BCR: B cell receptor; CNS: central nervous system; CSF: cerebral spinal fluid; DC: dendritic cell; EAE: experimental allergic encephalomyelitis; HLA: human leukocyte antigen; IgG, IgM: immunoglobulin G, M; Kiv: potassium inward rectifying channel; NMO: neuromyelitis optica; ON: optical neuritis; MOG: myelin oligodendrocyte glycoprotein; MS: multiple sclerosis; MG: Myasthenia Gravis; LETM: lateral extensive transverse myelitis; PDZ domain: post synaptic density protein, Drosophila disc large tumor suppressor, and zonula occludens-1 protein; SS: Sjögren syndrome; SLE: Systemic Lupus Erythematosus; TCR: T cell receptor; Th: T helper.

## Competing interests

The authors declare that they have no competing interests.

## Authors' contributions

WFW drafted the manuscript and helped edit and revise it, DJG generated the table and drafted the initial infectious disease section, ML made major contributions to the first rewrite and in particular the pathology section, DK made major contributions to the editing in particular, the overview of the pathology and pathogenesis of NMO. He also did the extensive proofing of the manuscript. All the authors read and approved the final version of the manuscript.
